# Gut mycobiome maturation and its determinants during early childhood: a comparison of ITS2 amplicon and shotgun metagenomic sequencing approaches

**DOI:** 10.3389/fmicb.2025.1539750

**Published:** 2025-05-21

**Authors:** Timothy Heisel, Sara Gonia, Abrielle Dillon, Susan L. Hoops, Gabriel A. Al-Ghalith, Daryl Gohl, Sagori Mukhopadhyay, Karen Puopolo, Peter Kennedy, Michael J. Sadowsky, Dan Knights, Abigail J. Johnson, Jeffrey S. Gerber, Cheryl A. Gale

**Affiliations:** ^1^Department of Pediatrics, University of Minnesota, Minneapolis, MN, United States; ^2^Department of Computer Science and Engineering, University of Minnesota, Minneapolis, MN, United States; ^3^Department of Soil, Water, and Climate, University of Minnesota Genomics Center, Minneapolis, MN, United States; ^4^Children’s Hospital of Philadelphia and University of Pennsylvania School of Medicine, Philadelphia, PA, United States; ^5^Department of Plant and Microbial Biology, University of Minnesota, St. Paul, MN, United States; ^6^Department of Soil, Water, and Climate, University of Minnesota, St. Paul, MN, United States; ^7^Biotechnology Institute, University of Minnesota, Minneapolis, MN, United States; ^8^School of Public Health, University of Minnesota, Minneapolis, MN, United States

**Keywords:** gut mycobiome, childhood, ITS2 amplicon, whole genome shotgun metagenomics, longitudinal variation

## Abstract

**Introduction:**

Microbial colonization of the gut in early life is important for the development of metabolism, immunity, and the brain. Fungi and bacteria both colonize the human infant gut. The relatively smaller contribution of fungi to the gut microbiome, as compared to bacteria, has posed technical challenges for the precise characterization of fungal communities (mycobiomes) and limited the ability to longitudinally examine mycobiome development.

**Background:**

The aims of this study were to (1) characterize mycobiome maturation and identify clinical determinants of mycobiome compositional variation during the first 2 years of life and (2) compare two sequencing approaches (ITS2 amplicon and whole genome metagenomics) for characterizing mycobiome maturational features. Longitudinal fecal samples and associated clinical metadata were obtained from subjects enrolled as part of the MAGIC (Microbiome, Antibiotics and Growth Infant Cohort) study.

**Results:**

Overall, fungal richness increased and mycobiome composition changed in a similar ordered pattern during the first 2 years of life utilizing either amplicon or metagenomic sequencing approaches. Less resolution of taxa to species and genera levels was observed for the metagenomic dataset. The predominant taxa identified by both sequencing approaches, *Candida albicans*, *Saccharomyces*/*S. cerevisiae*, and *Malassezia restricta*, each exhibited similar dynamics in abundances and prevalences over the first 2 years of life, irrespective of sequencing approach. Antibiotic exposure and breastfeeding status contributed to time-specific mycobiome compositional variation, results that were consistent for both types of sequence datasets. *Candida albicans* exhibited altered abundance dynamics in association with perinatal antibiotic exposure and birth mode for both sequencing approaches. *Post hoc* analyses suggested that the birth mode association could be driven by exposure to perinatal antibiotics in children delivered by cesarean section rather than by birth mode itself.

**Discussion:**

In summary, amplicon and metagenomic sequencing approaches provide generally similar results with respect to mycobiome maturational dynamics and the contribution of clinical variables to variation. Differences in taxa identification by the two approaches likely due to sequence database differences, primer/genome sequence variation, and/or sequencing depth should be taken into consideration.

## 1 Introduction

Fungi are the dominant eukaryotes in the human gut (Parfrey et al., 2014) and are present as commensal organisms, transient colonizers, and pathogens. Although the majority of preclinical studies have focused on gut bacterial communities (microbiomes) for their mechanistic role in host physiology, fungal communities (mycobiomes) have also been shown to contribute, in particular to the function of the immune system and metabolism (Iliev et al., 2012; Everard et al., 2014). In addition, a recent study in mice showed that gut fungi are causally implicated in the assembly of bacterial microbiomes (van Tilburg Bernardes et al., 2020). Together, these results in animal models have highlighted the importance of including fungi along with bacteria in studies aimed at elucidating the role of microbiomes in human health and disease.

In humans, obesity, inflammatory bowel disease, and some childhood allergies have all been associated with alterations in the gut mycobiome (Ott et al., 2008; Mar Rodríguez et al., 2015; Sokol et al., 2017; Boutin et al., 2021). It remains, however, unclear if and how mycobiomes and their interactions with microbiomes are mechanistically involved in the development of these diseases. It is particularly important to understand microbiome/mycobiome effects on health and disease during infancy, the time of life when gut microbes are co-developing with host aspects of physiology, with the potential then to affect short- as well as long-term health outcomes. For example, mice treated with low-dose antibiotics during infancy develop obesity and obesity-related metabolic phenotypes that persist into adulthood, despite normalization of their microbiomes as juvenile animals (Cho et al., 2012; Cox et al., 2014), and some studies report human infants with disrupted gut microbiomes due to antibiotic use have an increased incidence of being overweight and developing atopy and asthma later in life (Ajslev et al., 2011; Murphy et al., 2014; Mitre et al., 2018).

The majority of microbiome studies in human infants have used amplicon-based sequencing to characterize fungi, targeting 18S, ITS1, or ITS2 ribosomal DNA sequences. Disadvantages of amplicon-based approaches include primer bias, limited taxonomic resolution and challenges in differentiating between closely related species (Schoch et al., 2012; Lücking et al., 2020). In contrast, whole metagenome sequencing, a more widely used approach in studies of bacterial microbiomes (Stewart et al., 2018; Vatanen et al., 2018; Olm et al., 2022) provides sequence data from fragments of genes from all kingdoms of microbes and the host in a sample. This facilitates direct comparative analyses, within and between studies, of many types of organisms using one sequencing run. Advantages of metagenomic sequencing (reviewed in Nilsson et al., 2019) include the avoidance of biases inherent to PCR and primer choice, as well as the ability to assemble new genomes, distinguish closely related species from each other, and identify the presence of gene pathways toward the inference of microbiome function. A disadvantage of the metagenomic approach for characterization of fungi is the challenge in identification of low-abundance fungi, which historically has been less of an issue with the PCR amplicon approach. As sequencing costs have decreased, datasets are improving with respect to the depth of fungal sequencing, raising the potential for metagenomic sequencing to be a reasonable approach for mycobiome research.

In this study, we sought to characterize early life gut mycobiomes and their determinants and compare the results obtained with amplicon sequencing to those with whole genome metagenomic sequencing. Based on advantages and disadvantages being present for both sequencing approaches, we thought it would be important to understand the extent to which conclusions agreed and differed between approaches, to inform future studies. Overall, we found similar results with respect to general mycobiome development and its determinants over the first years of life when using either of sequencing approach. Differences in results were also observed, for example, in the identification as well as abundance dynamics of some fungal taxa.

## 2 Materials and methods

### 2.1 Participant information and sample collection and storage

Subjects were recruited as part of the “Microbiome, Antibiotic and Growth Infant Cohort” (MAGIC) study approved by the Committee for the Protection of Human Subjects at Children’s Hospital of Philadelphia (CHOP) (IRB 15-012623). This study was approved by the Human Research Protection Program at the University of Minnesota (U of MN) (IRB ID STUDY00003037) for the use of de-identified subject clinical and demographic data from CHOP and the use and generation of microbial sequence data from subject fecal samples. Informed consent was obtained from study subjects. The study enrolled children born at Pennsylvania Hospital (Philadelphia, PA) receiving preventive healthcare in the CHOP Primary Care Network or participating private practices together with their biological mothers. Demographic distributions of study participants (race, ethnicity, and sex assigned at time of birth) matched the general trends seen in the participating sites. Enrolled subjects were required to be less than 120 h of age, weigh more than 2,000 g, greater than 36 weeks gestational age, and admitted for less than 120 h to the neonatal care unit. All mothers were over the age of 18 years and spoke English. Maternal and child demographic and clinical data were obtained from questionnaires and medical chart reviews. For antibiotic covariable analyses, children who received antibiotics, up to and including the relevant sampling time point after birth, were compared to those who had not had postnatal antibiotic exposure. For maternal perinatal (near the time of birth) antibiotic covariable analyses, children born to mothers who received antibiotics were compared to those whose mothers did not. Breastfeeding status was defined as “yes” if the subject was breastfeeding some, or all, of their feedings at the time of sample collection. Reporting of race in this study cohort was mandated by the US National Institutes of Health, consistent with the Inclusion of Women, Minorities, and Children policy. Child race was reported by the mother via survey that included seven race categories (American Indian/Alaska Native, Asian, Black or African American, Native Hawaiian or Other Pacific Islander, White, more than one race, or unknown) and two ethnicity categories (Hispanic and non-Hispanic). For exploratory analyses, non-White subjects were grouped together (Group 1) for comparison to White subjects (Group 2), due to low numbers of non-white subjects.

Fecal samples were collected from participants and health questionnaires were completed within a few days of birth, and every 3 months until the subject reached 24 months of age. All subjects did not provide samples for every time point, and some samples were obtained at non-standard time points. Supplementary Figure 1 summarizes the timing and number of samples obtained by individual subject. Pea-sized volumes of fresh fecal samples from diapers were collected by healthcare providers or study personnel during the birth hospitalization and by parents at home after discharge using OMNIgene-GUT fecal collection kits (DNAgenoTek Inc., Ottawa, Ontario, CA). Collection tubes contain transport media that stabilizes DNA, allowing transport of samples at ambient temperatures.

### 2.2 DNA extraction, library creation, and sequencing

DNA was extracted from fecal samples using the DNeasy PowerSoil kit (QIAGEN, Germantown, MD, United States) by the University of Minnesota Genomics Center (UMGC). For amplicon sequencing, fungal ITS2 amplicons were generated from extracted DNA via synthesis of sequencing primers alongside amplicons (Gohl et al., 2016) in a dual-indexing method using a forward primer targeting the 5.8S fungal DNA region (Heisel et al., 2022) and reverse primer RSeq targeting the 25S region (Heisel et al., 2015). Amplicon libraries were sequenced using the Illumina MiSeq platform and 2 × 300 V3 chemistry. Eleven mock samples containing synthetic ITS sequences (Palmer et al., 2018) and 11 negative control (water) samples were also sequenced to evaluate for the presence of extraneous fungal DNA contamination during the amplicon sequencing process. Synthetic ITS sequences, DNA sequences that do not match any known fungal (or microbial) sequences, were used as controls to determine the extent of DNA spill-over into adjacent sequencing lanes and to determine the amount, if any, of microbial DNA contamination as compared to negative water control samples. For metagenomic sequencing, DNA libraries were constructed from extracted DNA using the Illumina Nextera XT ¼ kit (Illumina, San Diego, CA, United States). Metagenomic libraries were sequenced on an Illumina NovaSeq, using the S4 flow cell with the 2 × 150 bp paired end V4 chemistry kit, and achieved a median sequencing depth of ~4.5 million total sequence reads per sample. Overall, fungal sequence reads were present in fecal samples at a median of one in every 2.2 million total metagenomic sequence reads, in general agreement with a prior study of infant mycobiomes [1 fungal read for every 1.4 million total reads (Auchtung et al., 2022)].

### 2.3 Sequence processing and taxonomy assignment

Resulting sequences in FASTQ format were stored and processed on hardware available through the Minnesota Supercomputing Institute. Raw FASTQ files from both the amplicon and shotgun metagenomic sequencing runs were initially processed individually through Shi7 (Al-Ghalith et al., 2018) version 0.9.9. Folders containing the raw sequence files were presented to shi7_learning.py, and the output from shi7_learning.py was used to inform and process the samples using shi7.py to generate quality controlled, trimmed, and multiplexed FASTA files (commands for both ITS and metagenomics: shi7_learning.py -i ./fastq -o learnt, shi7.py -i ./fastq -o ./output --adaptor Nextera). For metagenomic sequences, 50 GB of memory and 24 processing cores were provided to shi7_learning.py to process four folders of approximately 1 TB of raw FASTQ files each; the time required for this shi7_learning step was several hours. For processing the metagenomic sequence files through shi7.py, a supercomputer node containing 900 GB of memory and 24 processing cores was used; processing generally required between 24 to 36 h to complete. Due to memory constraints, the metagenomic sequences were divided into four batches for processing by shi7, with four subsequent FASTA files generated. In contrast, the amplicon sequences required much less computational hardware and time for the generation of FASTA files and were able to be processed on an interactive terminal using desktop equivalent amounts of memory and processing power in a matter of minutes. Sequences remaining after processing via shi7 were then aligned to reference databases using BURST (Al-Ghalith and Knights, 2017) version 0.99. To align amplicon sequences, a reference database was generated from the ITS RefSeq collection compiled by the National Center for Biotechnology Information (NCBI) (Heisel et al., 2022) (command for ITS reads and BURST: burst12 -q combined_taxa.fna -a ITS_20180808.acx -r ITS_20180808.edx -b ITS_20180808.tax -o fungal_its.b6 -fr -t 24). The amplicon database contains the rRNA/ITS region from 8,902 fungal species. Sequences corresponding to the synthetic ITS controls were added to the amplicon database before use. To align metagenomic sequences, a database was generated from the RefSeq collection of reference fungal genomes compiled by the NCBI, accessed on February 17, 2020 (command for metagenomic reads and BURST: burst15 -q combined_taxa.fna -a Fungus02172020.acx -r Fungus02172020.edx -b Fungus02172020.tax -o fungal_wgs.b6 -fr -t 24). The fungal metagenomic database contains the full genome sequences from 301 fungal species. Manual curation of this database was required, as numerous genomes contained spurious contigs (i.e., sequences that were identified as either bacterial or human DNA by comparing suspicious contigs to the “nr” NCBI database using NCBI-BLAST), as previously noted by other researchers (Lind and Pollard, 2021). Contigs were identified as suspicious if they were found to align alongside non-fungal taxonomies when analyzing sequencing data using the -m ALLPATHS flag in BURST with a comprehensive bacterial and fungal RefSeq BURST database. For both sequencing approaches, alignment of sequences utilized BURST with a 95% identity cutoff flag with the forward/reverse complement flag activated. Processing the 9.4 GB amplicon FASTA file required significantly less computational power than the 200 GB metagenomic FASTA files through BURST (Amplicon: 60 GB of memory, 24 processing cores, approximately 3 h of time, Metagenomics: 990 GB of memory, 24 processing cores, with each of the 4 metagenomic sequencing files taking approximately 2.5 days of time). The resulting .b6 files were converted to reference and taxonomy tables using embalmulate (Al-Ghalith and Knights, 2017) with “GGtrim” activated for both sequence datasets (command for ITS reads and embalmulate: embalmulate fungal_its.b6 fungal_its.txt fungal_its_tax.txt “GGtrim,” command for metagenomic reads and embalmulate: embalmulate fungal_wgs.b6 fungal_wgs.txt fungal_wgs_tax.txt “GGtrim”).

### 2.4 Sequence analyses and data visualization

Taxonomy and metadata tables were imported into RStudio for analysis. The amplicon taxonomy table and the four metagenomic taxonomy tables, were pooled into a single metagenomic taxonomy table and subjected to quality control screening as follows. Sequence read counts were determined for each sample, and those samples residing in the lowest quartile of read counts were deemed to be potentially unrepresentative of the population due to their rarity and were discarded. The resulting sequence read cutoff value per sample was 39 for amplicon, and 69 for metagenomic sequencing approaches. Thus, the number of samples remaining (after quality control processes) for subsequent analyses was 773 for the amplicon sequence dataset and 756 for the metagenomic sequence dataset. To eliminate rare or spurious taxa, any taxon that was not present in at least five of the total number of samples for each sequencing approach was removed. Sequence data was normalized using either centered log ratio (CLR) transformation (for beta diversity determined by Euclidean distance) or median depth normalization (for taxa abundances). Taxonomic sequence data was transformed into proportionally transformed percentile abundances (for amplicon data) or actual abundances (for metagenomic data). Taxa counts were log transformed to convert results into normal distributions prior to statistical analyses. The diversity and specnumber functions in the vegan R package and lme function in the nlme R package were used to calculate and analyze alpha diversities (taxa count and Shannon diversity index were used). Taxa abundances over time were modeled using LOESS (LOcally Estimated Scatterplot Smoothing) regression and plotted with 95% confidence intervals in RStudio to visualize taxa abundance dynamics, and linear mixed-effects models were used to assess differences over time. Beta diversity analyses (PERMANOVAs) were performed using the pairwise Adonis function in the vegan/BiodiversityR packages in R. Data manipulations and visualizations were achieved with the following computational packages: ggplot2, ggbeeswarm, ggsignif, plyr, dplyr, tidyr, textshape, reshape2, and randomcoloR. To account for the possibility of false positive results due to multiple tests in the case of EnvFit analyses, *p*-values were adjusted using false discovery rate (FDR) correction with “FDR-corrected *p*” (*q*) < 0.25 considered statistically different. For longitudinal analyses of alpha diversity using the entire sample set, multiple sampling of subjects was corrected for by including subject ID as a random effect in statistical models. For longitudinal analyses of mycobiome beta diversity, samples were grouped into seven discrete “time clusters” (determined on the basis of similar numbers of samples in each grouping) according to the age of the subject at sample collection: cluster 1, 0–4 weeks; cluster 2, 5–13 weeks (2); cluster 3, 14–24 weeks; cluster 4, 25–47 weeks; cluster 5, 48–50 weeks; cluster 6, 51–63 weeks; cluster 7, 64–96 weeks. Only the first (“youngest”) sample from a given subject was included for each time cluster to control for multiple sampling (Stewart et al., 2018) (also noted in the text, tables, or figures where appropriate). Covariable contributions to mycobiome variation (beta diversity) for each time cluster were determined via the EnvFit function in RStudio. CLR-normalized fungal sequence data, with beta diversity distances determined by the Euclidean calculator, were used in EnvFit analyses for both the amplicon and metagenomic datasets, with statistical values generated from comparisons of the differences between variable centroids relative to the total variation in microbial beta diversity for each time cluster. We considered *p*-values <0.05, and FDR-corrected *p* (*q*)-values of <0.25 as statistically significant. Taxon-level associations with clinical variables that have *q*-values <0.25 are considered statistically significant in exploratory microbiome studies where further investigation and validation of results are necessary (Stewart et al., 2018; Stražar et al., 2021; Martin et al., 2022). In addition, for mycobiome comparisons between demographic and clinical groups (e.g., vaginal versus cesarean-section birth), potential covariables were added into the statistical models and, for the majority, did not contribute to significant associations between the primary variable with mycobiome variation, except where noted in the Results section.

## 3 Results

### 3.1 Study population characteristics

Overall, 773 fecal samples provided amplicon sequences, 756 samples provided metagenomic sequences, and 590 samples provided both types of sequence data for this study (after sequencing quality control measures were performed), which represented samples from 177 children (distribution of samples over time is shown in Supplementary Figure 1). Of these subjects, approximately 63% were delivered vaginally, 48% were female, 70% identified as White, and 54% were exposed to perinatal antibiotics. The mean gestational age at birth for the study cohort was 39.7 weeks (SD ± 1.1). Most of the children in this study (86%) had a birth weight between the 10th and 90th percentiles; 9% were below the 10th percentile and 6% were above the 90th percentile. Of the covariables considered in this study, only breastfeeding status and childhood antibiotic exposure significantly differed by subject age. The percentage of children engaged in breastfeeding significantly decreased over time from >95% during the first month of life to ~30% between 16–24 months of age, whereas those exposed to childhood (postnatal) antibiotics increased over time, from a low of <3% during the first month of life and reaching a plateau of ~50–60% in children over 6 months of age (Table 1).

**Table 1:** Comparison of covariables by time of sample collection^a^.

Amplicon dataset								
	Cluster 1 (90)^b^	Cluster 2 (38)	Cluster 3 (67)	Cluster 4 (67)	Cluster 5 (84)	Cluster 6 (74)	Cluster 7 (66)	*p*-value^c^
Time range (weeks postpartum)	0–4	5–13	14–24	25–47	48–50	51–63	64–96	n/a
Perinatal antibiotics (% exposed)	55.6	68.4	59.7	52.2	60.0	52.7	47.0	0.48
Childhood antibiotics (% exposed)	2.2	13.2	16.4	56.7	51.2	56.8	59.1	<0.001
Breastfeeding status (% yes)	97.8	89.5	83.6	71.6	58.3	35.1	27.3	<0.001
Infant race (% Group 2)^d^	67.8	68.4	77.6	79.1	72.6	78.4	78.8	0.50
Infant sex (% male)	48.9	52.6	56.7	58.2	50.0	55.4	60.1	0.76
Birth mode (% vaginal)	58.9	39.5	55.2	56.7	57.1	58.1	68.2	0.21

Metagenomic dataset								
	Cluster 1 (106)^b^	Cluster 2 (46)	Cluster 3 (68)	Cluster 4 (62)	Cluster 5 (76)	Cluster 6 (64)	Cluster 7 (55)	*p*-value^c^
Time range (weeks postpartum)	0–4	5–13	14–24	25–47	48–50	51–63	64–96	n/a
Perinatal antibiotics (% exposed)	52.8	67.4	58.8	54.8	59.2	51.6	49.1	0.54
Childhood antibiotics (% exposed)	0.94	13.0	19.1	56.5	53.9	54.7	60.0	<0.001
Breastfeeding status (% yes)	97.2	89.1	88.2	74.2	57.9	39.1	30.9	<0.001
Infant race (% Group 2)^d^	67.9	67.4	79.4	80.6	75.0	81.3	80.0	0.23
Infant sex (% male)	47.2	54.3	57.4	59.7	52.6	57.8	60.0	0.72
Birth mode (% vaginal)	59.4	43.5	54.4	53.2	55.3	59.4	67.3	0.34

a Only the earliest sample in each time cluster per infant was included in these analyses to control for multiple sampling effects.

b (Sample *n*).

c Comparison of covariable groups among all time clusters was performed using Kruskal–Wallis tests.

d See Supplementary Table 1 for breakdown of specific race groups.

### 3.2 Identification of fecal fungi and comparison of amplicon to metagenomic sequencing approaches

Amplicon and metagenomic sequencing of fecal DNA identified 197 and 253 fungal taxa respectively; of these, 37 taxa were identified by both sequencing approaches. However, these common taxa comprised most of the abundant taxa with a combined abundance of 75% in the amplicon dataset and 60% in the metagenomic dataset. For either sequencing approach, the most abundant taxa were generally observed to also be the most prevalent for the cohort (Supplementary Table 2). Across both sequence datasets, the major fungal taxa in common were *Candida albicans*, *Saccharomyces*/*Saccharomyces cerevisiae*, *Malassezia restricta*, and *Candida tropicalis*. The amplicon dataset contained more unique *Candida* species whereas the metagenomic dataset contained more unique *Aspergillus* species. Of note, *Candida parapsilosis*, a common gut commensal and cause of infection during infancy, was identified as highly abundant and prevalent in this cohort, but only by the amplicon sequencing approach. In general, less resolution of taxa to species and genera levels was observed in the metagenomic dataset (96% of taxa for amplicon dataset vs. 89% of taxa for metagenomic dataset).

### 3.3 Gut mycobiomes are dynamic over the first 2 years of life

**Figure 1:**
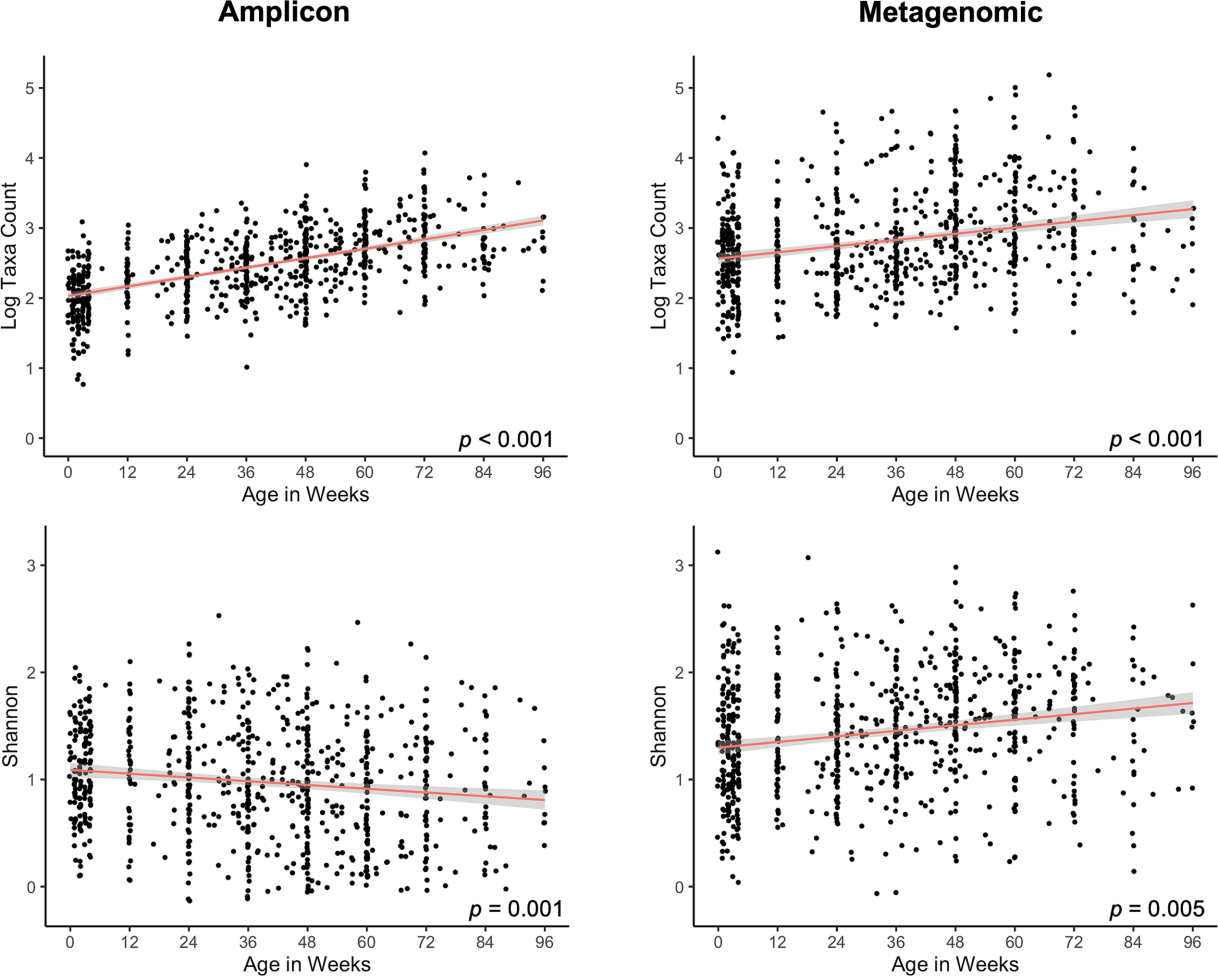
Dot plots of fungal alpha diversity over the first ~2 years of life using amplicon (left panels) and metagenomic (right panels) sequences. Alpha diversity measures: richness (top panels), Shannon index (bottom panels). Each dot represents an individual sample, and all samples are organized by time of collection as a continuous variable. Random sampling was used in linear statistical models to account for multiple samples per infant. Linear regression line of best fit, red; gray shading, 95% confidence intervals.

**Figure 2:**
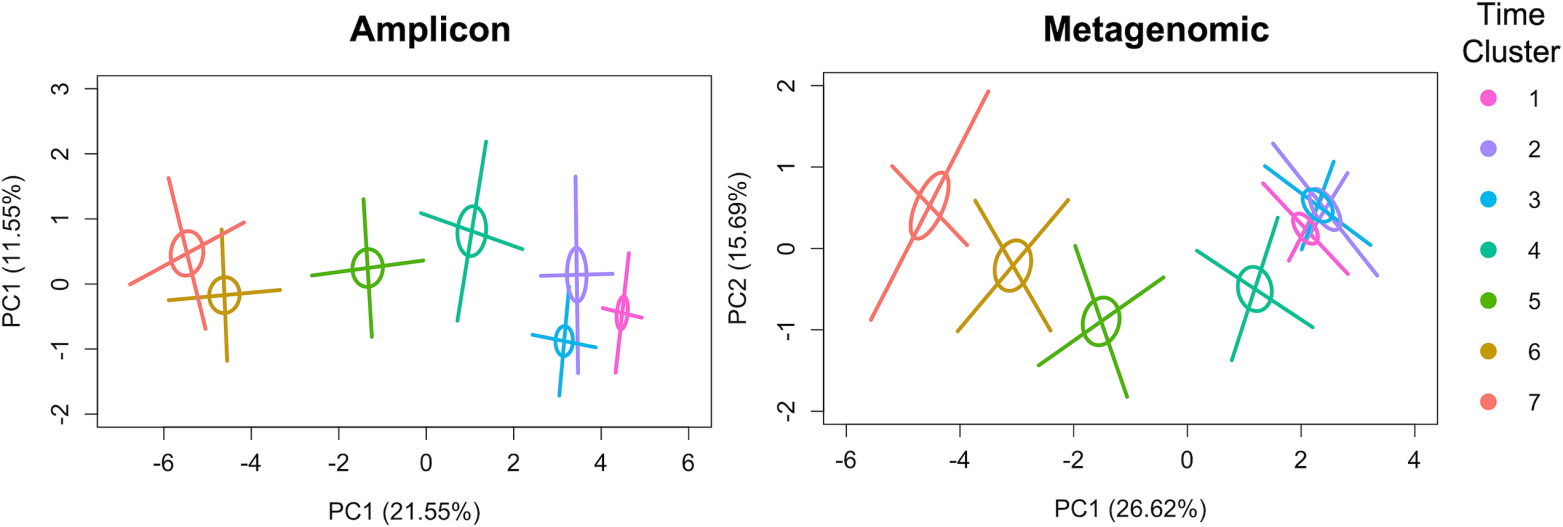
Principal coordinates analysis (PCoA) plots of fecal fungal taxonomic compositions (beta-diversity) by time cluster and sequencing approach. Ellipses show the standard error (SE) confidence limit set at 0.2, and crosshatches show the standard deviation (SD) with confidence limit set at 0.95. In the case of multiple samples from an infant in a time cluster, only the earliest sample was used in the analysis. Statistical comparisons of fungal beta diversity among time clusters and number of samples (*n*) within each time cluster for each sequencing dataset are found in Supplementary Table 4.

Fungal diversity features in the gut had similar dynamics over time in the children in this cohort, irrespective of sequencing dataset analyzed except for one feature, as discussed below. Fungal richness (taxa numbers) increased over time (Figure 1) in both datasets. Shannon diversity indices also increased over time, but only for the metagenomics dataset. The finding that Shannon indices had an opposite (decreasing) trend over time in the amplicon dataset suggests that there is a strong decline in evenness of fungal taxa abundances over time in this sequence dataset (Figure 1). In addition, fecal fungal community compositions (beta diversities) were significantly different for most of the pairwise comparisons between time clusters for both sequencing datasets (Supplementary Table 3). These mycobiome compositional dynamics are visualized in PCoA plots, with beta-diversity centroids moving from right to left in a generally ordered pattern as subject age increases and more heterogeneity in mycobiomes being observed among later time clusters, for both sequencing datasets (Figure 2). The finding that beta diversity did not differ between the time clusters corresponding to the oldest ages (clusters 6 and 7), for either sequencing dataset, may indicate that mycobiome composition is reaching a maturational plateau (Supplementary Table 3).

**Figure 3:**
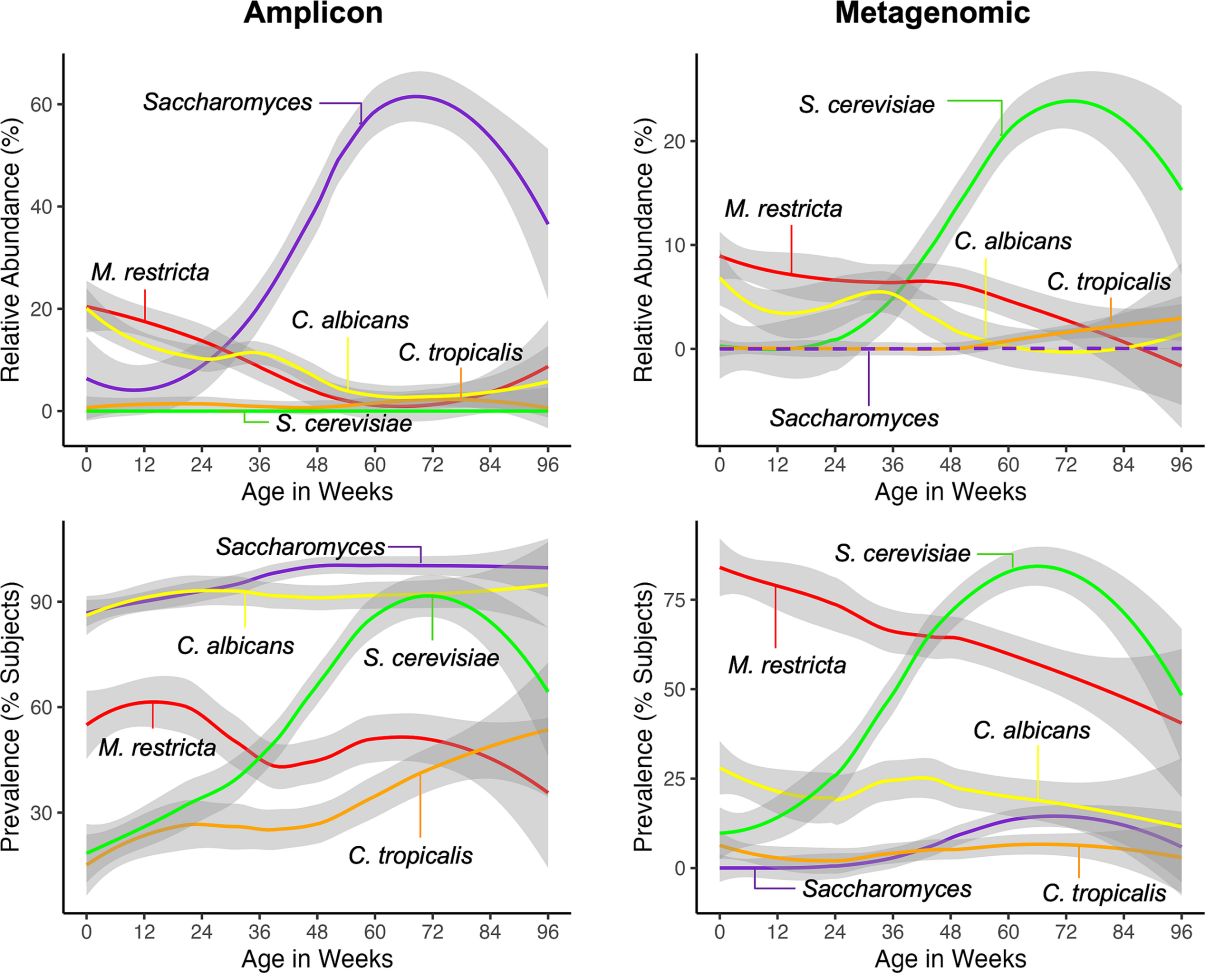
LOESS regression curves of relative abundances and prevalences of dominant fungal taxa during the first 2 years of life for amplicon and metagenomic sequence datasets. LOESS regression curves for individual taxa indicated by different colors; gray shading, 95% confidence intervals. Dashed line not detected but included for comparison.

We also analyzed the relative abundances and prevalences of the prominent fungal taxa identified by both sequencing datasets. We observed that *C. albicans*, *Saccharomyces*/*S. cerevisiae*, and *M. restricta* had similar abundance and prevalence dynamics by either sequencing approach (Figure 3 and Supplementary Table 4). The abundance of *C. albicans* decreased and prevalence remained stable, *Saccharomyces*/*S. cerevisiae* abundance and prevalence increased, and *M. restricta* abundance and prevalence decreased (Figure 3). Although the abundance of *C. tropicalis* was generally stable over time by both sequencing approaches, its prevalence dynamics were not similar for amplicon and metagenomic datasets.

### 3.4 Contributions of clinical and demographic variables to early life mycobiome compositional variation and comparison by sequencing approach

#### 3.4.1 Alpha and beta diversity

As previously noted, mycobiome richness increased over the first 2 years of life, irrespective of the sequence dataset used. To understand if clinical and demographic variables were associated with differences in alpha diversity measures at specific times, we analyzed mycobiome richness and Shannon indices by clinical factor and for each individual time cluster (Supplementary Table 5). No variables consistently (across all time clusters) differed for either alpha diversity metric. Although some differences were observed for individual time clusters, these were not consistent across both sequence datasets. To examine the extent to which clinical and demographic variables explain the variance in mycobiome compositions (beta diversity) during early life, we performed EnvFit analysis by time cluster for both amplicon and metagenomic datasets. EnvFit reports the significance (*p*-value, comparing all variables within each time cluster) and amount of variance (*r*^2^) explained by each covariable in the statistical model.

**Figure 4:**
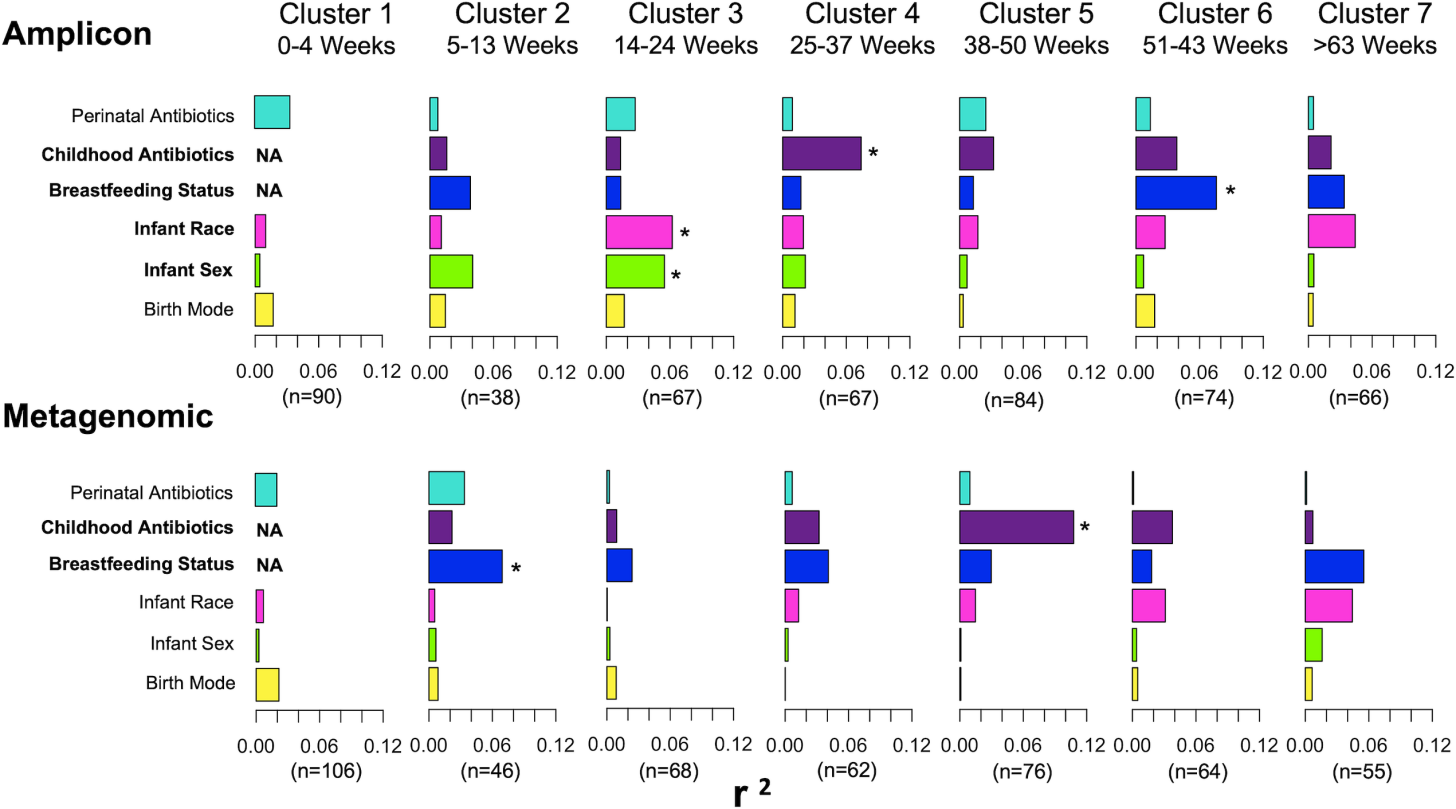
Bar plots of explained variance of six potential mycobiome covariables modeled by EnvFit for amplicon and metagenomic sequence data. Horizontal bars represent the amount of variance (*r*^2^) explained by each covariable in the model. Covariables that contributed significant differences in mycobiome composition (*p*-value <0.05 with *q*-value of <0.25) are represented in bold font. Asterisk denotes statistical significance within a time point. For amplicon dataset: time cluster 3, infant race (*p* = 0.017, *q* = 0.05) and infant sex (*p* = 0.015, *q* = 0.05); time cluster 4, childhood antibiotics (*p* = 0.005, *q* = 0.03); time cluster 6, breastfeeding status (*p* = 0.027, *q* = 0.16). For metagenomic dataset: time cluster 2, breastfeeding status (*p* = 0.039, *q* = 0.23); time cluster 5, childhood antibiotics (*p* = 0.001, *q* = 0.006). NA, not able to perform statistical analysis due to inadequate number of subjects within one of the comparison groups (e.g., not breastfeeding and exposed to childhood antibiotics for time cluster 1).

Several variables were observed to contribute to beta diversity consistently across both sequencing approaches for some time clusters. For the earliest time (cluster 1), perinatal antibiotic exposure and birth mode explained the greatest amount of mycobiome variation, as compared to the other variables (note relative sizes of bars on Figure 4). Breastfeeding status was also a major contributor to variation at early times (cluster 2) for both sequencing datasets. At later times (clusters 4–6), childhood antibiotic exposure was the predominant consistent variable explaining mycobiome variation, whereas for the latest time (cluster 7), the greatest amount of mycobiome variation was explained by breastfeeding status and race.

#### 3.4.2 Fungal taxa abundances

Several clinical variables were associated with differences in the abundance dynamics of the predominant fungal taxa (featured on Figure 3) in the feces of the study cohort during the first 2 years of life. Variables that showed significant associations with taxa abundance differences over time for both sequencing approaches were childhood antibiotic exposure, breastfeeding status, perinatal antibiotic exposure and birth mode (Supplementary Table 6). Childhood antibiotic exposure was associated with differences for *Saccharomyces*/*S. cerevisiae* abundance dynamics, with exposure being associated with generally higher abundances of *Saccharomyces*/*S. cerevisiae* (positive beta-coefficient, Supplementary Table 6), in particular at <6 months of age (Supplementary Figure 2). Breastfeeding was associated with differences in *C. albicans* (higher, positive beta-coefficient) as well as S*accharomyces*/*S. cerevisiae* (lower, negative beta-coefficient) abundances over time (Supplementary Table 6), but the dynamics differences were not completely consistent between the two sequencing approaches (Supplementary Figure 2). Exposure to perinatal antibiotics was associated with higher *C. albicans* abundances throughout the time course (positive beta-coefficient, Supplementary Table 6 and Supplementary Figure 2), however, this association did not remain significant when birth mode was added into the statistical model. Similarly, *C. albicans* abundances tended to be higher over time in children delivered by cesarean section (positive beta-coefficient, Supplementary Table 6 and Supplementary Figure 2), but the difference did not remain significant when the perinatal antibiotic exposure variable was included in the statistical model. The interaction between birth mode and perinatal antibiotics aligns with the fact that antibiotics were administered to all mothers at the time of delivery of their infants by cesarean section. To attempt to separate the effects of perinatal antibiotics from those of birth mode, *C. albicans* abundances over time were compared by birth mode in the subset of infants exposed to perinatal antibiotics. In this comparison, no difference in *C. albicans* abundance dynamics were observed between birth mode groups (linear model, *p* > 0.05), which suggests that perinatal antibiotics may be the major driver of *C. albicans* abundance variation observed in children delivering by cesarean section in this cohort.

## 4 Discussion

Metagenomic sequencing, as compared to amplicon-based sequencing, has not been widely used to characterize fungal communities in humans, and only rarely in infant cohorts. The reasons for this have included the lower amounts of fungi (compared to bacteria) in human microbiomes, the higher costs for increased depth of sequencing to capture fungi, and less coverage of fungi in metagenomic sequence databases. In a recent study comparing amplicon (ITS2) and whole genome metagenomic sequencing approaches for the study of gut mycobiomes in healthy adults, general concordance was observed with respect to the identification of fungi, with *Candida*, *Saccharomyces*, and *Malassezia* species being among the most abundant fungal taxa (Nash et al., 2017). The detailed analysis of mycobiome longitudinal dynamics, and clinical determinants, however, was not explored and compared between approaches. With continued technological improvements and lower costs facilitating increased depth of sequencing, whole genome metagenomic sequencing may, in some cases, be a feasible strategy allowing the comparison of multiple microbial kingdoms, simultaneously with the genome of the host, using a single sequencing approach. The resultant understanding of cross-kingdom interactions would ultimately inform a more complete mechanistic picture of how microbiomes are involved in the development of human health and disease.

Prior studies of mycobiome development in infants have primarily utilized an amplicon sequencing approach, targeting ITS1 and ITS2 regions of the fungal rDNA locus, to characterize fungal communities (Heisel et al., 2015; Boutin et al., 2021; Auchtung et al., 2022; Gutierrez et al., 2023). Recently, two studies reported the use of metagenomic sequencing to characterize features of infant gut mycobiomes (Lind and Pollard, 2021; Auchtung et al., 2022). Altogether, studies utilizing either sequencing approach have found that *Candida* and *Saccharomyces* species were the major fungal taxa identified in the feces of young children. Consistent with these prior reports, we also found that amplicon and metagenomic sequencing approaches identified *Candida* and *Saccharomyces* species as predominant fecal taxa during early life. In the comparison of the two sequencing approaches for their ability to identify fungi, we found that ~20% of the total identified sequences were shared between the sequencing approaches. The taxa that were identified by both methods, however, comprised 60% of the fungal sequences present in the metagenomic dataset and 72% of the sequences in the amplicon dataset. Thus, although differences exist with respect to the identification of less common fungi, amplicon and metagenomic sequencing approaches have the potential to provide consistent results with respect to predominant mycobiome features and their variation.

Overall, we found general consistency between amplicon and metagenomic sequencing approaches with respect to how gut mycobiome features develop over the first years of life. Fungal richness increased over time and variation in fungal compositions (beta diversity) occurred with a similar ordered pattern for both sequencing approaches. The findings that mycobiome diversity and composition are dynamic and change during early life is a theme echoed by several previous studies utilizing either amplicon or metagenomic sequencing approaches (Fujimura et al., 2016; Schei et al., 2017; Wampach et al., 2017; Amenyogbe et al., 2021; Boutin et al., 2021; Auchtung et al., 2022; Gutierrez et al., 2023). In addition, we observed similar abundance and prevalence trends for the major fungal taxa *C. albicans*, *Saccharomyces*/*S. cerevisiae*, and *M. restricta* during early life, irrespective of sequencing approach, and are consistent with abundance dynamics reported by others (Lind and Pollard, 2021; Auchtung et al., 2022; Gutierrez et al., 2023).

Previous studies have observed that antibiotic exposure is a major contributor to gut bacterial compositional variation (Dethlefsen et al., 2008; Antonopoulos et al., 2009; Robinson and Young, 2010; Dethlefsen and Relman, 2011). In the current study, perinatal and childhood antibiotic exposure were major clinical variables associated with mycobiome feature variation over the first years of life. The result that antibacterial antibiotics have a trans-kingdom effect on fungal communities is consistent with the findings of other studies of gut mycobiomes (Heisel et al., 2022; Ventin-Holmberg et al., 2022). In the Ventin-Holmberg et al. (2022) study, antibiotic exposure in infants was associated with lower bacterial diversity and higher fungal diversity and abundances of several fungi, including *C. albicans*. This supports the idea that antibacterial antibiotics modulate mycobiomes via disruption of the normal balance of the bacterial community. Perinatal antibiotic exposure contributed more to mycobiome variation (beta diversity) during early times after birth, with generally higher abundances of *C. albicans* not only at early times but also over the entire time course. This result appears to be independent of birth mode. We did not have access to information about the reasons for perinatal antibiotic use, thus, we cannot rule-out the possibility that additional covariables contributed to this result. In contrast to perinatal antibiotics, childhood antibiotic exposure explained more of the compositional variation in mycobiomes at older ages and was associated with generally higher abundances of *Saccharomyces*/*S. cerevisiae* over time, although differences in *Saccharomyces*/*S. cerevisiae* abundances were not completely consistent across sequencing approaches. The finding that increased *S. cerevisiae* abundances may be associated with antibacterial antibiotic exposure is consistent with a recent report that broad spectrum antibiotic administration to mice enhances their ability to be colonized with *Saccharomyces* species in the gut (Hedin et al., 2022).

Our study, along with those of other investigators (Boutin et al., 2021; Auchtung et al., 2022), found that breastmilk feeding is associated with mycobiome composition during early childhood. Breastmilk is a complex, dynamic fluid that contains thousands of distinct components including nutrients and non-nutritive “bioactive” components [human milk oligosaccharides (HMOs), immune cells, bacteria and fungi] that promote healthy immune, metabolic and organ development (Bardanzellu et al., 2020). Milk nutrients may affect mycobiome compositions directly via the provision of nutrients that favor specific fungi over others. Bioactive components like HMOs, which promote the increased abundances of probiotic bacteria (Lactobacillus, Bifidobacteria) in the infant gut, and milk bacterial microbiomes themselves, could affect mycobiome compositions indirectly via interkingdom (bacterial-fungal) or immune cell-mediated interactions (van Tilburg Bernardes et al., 2020). Breastmilk fungi, the presence of which has been reported by several investigators (Heisel et al., 2019; Boix-Amoros et al., 2017; Moossavi et al., 2020), is a potential direct source of fungi for developing infant gut mycobiomes. Overall, much work remains to be done to determine how breastmilk components are inter-related with each other and with the infant gut in modulating early life microbiome development.

We acknowledge that our comparison of sequencing approaches is limited by the sequencing depths chosen, particularly for metagenomic sequencing. While the depth of metagenomic sequencing in the current study is similar to that reported in the study of infant gut microbiomes by Auchtung et al. (2022), approximately 1–2 gigabases/sample, it is ~10-fold less than that reported by the Human Microbiome Project study of adult gut microbiomes (Nash et al., 2017). With respect to PCR-based amplicon sequencing, this approach did not detect some taxa that the metagenomics approach did in our study. This result may be due, in part, to sequence mismatches between the ITS2 primers and the sequences of some fungal taxa, resulting in sub-optimal amplification. Even though ITS2 sequence databases include many more fungal taxa than metagenomic databases, it is also possible that they contain gaps in coverage. Finally, we did not include a host depletion step (either pre-sequencing or during the bioinformatics process) for the metagenomics process, thus, there is a chance that a small number of human reads identical to a reference fungal sequence could align to the fungal reference database, reducing the accuracy of our final metagenomic dataset. On the other hand, inclusion of a host sequence depletion step could also result in the rejection of true fungal sequences that align with regions of conservation in the host reference sequence database. Despite these considerations, this study has several strengths. A large infant cohort with a high number of longitudinal samples is represented in the analysis. In addition, we obtained high quality antibiotic exposure data for the entire cohort (duration and timing of exposure). This was due to collaborations with the Children’s Hospital of Philadelphia Health Network, the nation’s largest integrated pediatric health care system that provides electronic medical records connecting hospital and clinic data for children from birth onward and the Department of Biomedical and Health Informatics, which tracks office- and telephone-based prescriptions for study subjects and shares a common electronic health record with the CHOP Health Network. The quality of longitudinal antibiotic exposure data in this study may have allowed us to observe associations between antibiotics and early life mycobiome features that other studies have not.

In summary, our study supports the potential for metagenomic sequencing to be a useful approach for the characterization of gut mycobiomes and the identification of factors that contribute to fungal community variation. Higher sequencing depths and further improvement toward more comprehensive fungal sequence databases will continue to increase the robustness of mycobiome characterization in the future. Amplicon sequencing may be advantageous in situations where identification of less abundant fungi is important to the research question, whereas a metagenomics approach could be advantageous toward the study of interkingdom relationships and their impact on health outcomes using a single sequencing experiment.

## Data Availability

Raw sequence files can be found at the NCBI bio project https://www.ncbi.nlm.nih.gov/bioproject/PRJNA903921/. ITS and metagenomic fungal sequence databases can be found at: https://zenodo.org/records/15263682. Metadata and RStudio processing files can be found at: https://github.com/kegle015/Gut-Mycobiome-Maturation-and-Its-Determinants-During-Early-Childhood.
