# Supplementary material for: Gut mycobiome maturation and its determinants during early childhood: a comparison of ITS2 amplicon and shotgun metagenomic sequencing approaches
Source: Front Microbiol. 2025 May 21;16:1539750. doi: 10.3389/fmicb.2025.1539750 (PMC12133761; doi:10.3389/fmicb.2025.1539750)
Supplement: Supplementary file 1 [file Table_1.docx]

Supplementary Material

# Supplementary Tables

## 2.1 Supplementary Table 1. Number of subjects for each race group by time cluster of sample collection^1^

**Amplicon dataset**

|  | Cluster 1 | Cluster 2 | Cluster 3 | Cluster 4 | Cluster 5 | Cluster 6 | Cluster 7 |
| --- | --- | --- | --- | --- | --- | --- | --- |
| American Indian | 1 | 0 | 0 | 0 | 0 | 0 | 0 |
| Asian | 3 | 1 | 0 | 3 | 2 | 2 | 2 |
| Black or African American | 8 | 3 | 3 | 3 | 5 | 3 | 4 |
| More than one race | 17 | 8 | 11 | 8 | 14 | 9 | 7 |
| White | 61 | 26 | 52 | 50 | 59 | 56 | 51 |

**Metagenomic dataset**

|  | Cluster 1 | Cluster 2 | Cluster 3 | Cluster 4 | Cluster 5 | Cluster 6 | Cluster 7 |
| --- | --- | --- | --- | --- | --- | --- | --- |
| American Indian | 0 | 0 | 0 | 0 | 0 | 0 | 0 |
| Asian | 5 | 1 | 1 | 3 | 2 | 1 | 1 |
| Black or African American | 10 | 3 | 3 | 1 | 6 | 2 | 4 |
| More than one race | 19 | 11 | 10 | 8 | 11 | 9 | 6 |
| White | 72 | 31 | 54 | 50 | 57 | 52 | 44 |

^1^Only the earliest sample in each time cluster per infant was included in these analyses to control for multiple sampling effects.

## 2.2 Supplementary Table 2. Top 10 taxa across sequencing methods

|  | ITS2 Analysis | | | | Metagenomic Analysis | | | |
| --- | --- | --- | --- | --- | --- | --- | --- | --- |
| Taxa | % Subjects | Rank | % Reads | Rank | % Subjects | Rank | % Reads | Rank |
| ***g__Saccharomyces*¹** | 95.86 | 1 | 44.62 | 1 | 5.16 | 102 | 0.04 | 58 |
| ***s__Candida albicans*** | 91.20 | 2 | 21.31 | 2 | 22.49 | 19 | 13.97 | 2 |
| *s__Candida parapsilosis* | 74.00 | 3 | 8.01 | 3 | N.D. | N.D. | N.D. | N.D. |
| *s__Cladosporium ossifragi* | 62.23 | 4 | 4.93 | 4 | N.D. | N.D. | N.D. | N.D. |
| ***s__Candida tropicalis*** | 28.85 | 12 | 3.39 | 5 | 4.76 | 106 | 0.10 | 32 |
| *s__Rhodotorula mucilaginosa* | 40.88 | 9 | 1.58 | 6 | N.D. | N.D. | N.D. | N.D. |
| ***s__Malassezia restricta*** | 51.23 | 6 | 1.35 | 7 | 69.58 | 5 | 1.08 | 13 |
| *s__Candida sake* | 17.85 | 15 | 1.08 | 8 | N.D. | N.D. | N.D. | N.D. |
| ***s__Debaryomyces hansenii*** | 15.65 | 19 | 1.04 | 9 | 2.38 | 164 | 0.02 | 82 |
| *s__Filobasidium magnum* | 14.23 | 22 | 0.81 | 10 | N.D. | N.D. | N.D. | N.D. |
| ***s__Saccharomyces cerevisiae*** | 54.20 | 5 | 0.35 | 21 | 44.31 | 10 | 32.17 | 1 |
| *s__Aspergillus niger* | N.D. ^2^ | N.D. | N.D. | N.D. | 84.26 | 3 | 13.38 | 3 |
| ***s__Aspergillus flavus*** | 1.16 | 129 | <0.01 | 137 | 91.53 | 1 | 9.82 | 4 |
| *s__Aspergillus clavatus* | <0.01 | 452 | <0.01 | 313 | 56.75 | 6 | 4.05 | 6 |
| *s__Sordaria macrospora* | N.D. | N.D. | N.D. | N.D. | 56.48 | 7 | 3.00 | 7 |
| *s__Grosmannia clavigera* | N.D. | N.D. | N.D. | N.D. | 49.60 | 9 | 1.61 | 9 |
| *s__Aspergillus fischeri* | N.D. | N.D. | N.D. | N.D. | 15.74 | 36 | 1.37 | 10 |

^1^Bold font highlights the taxa identified by both sequencing approaches.

^2^N.D. Not detected

## 2.3 Supplementary Table 3. Comparison (q-values) of fecal fungal beta diversity between time clusters^1^

**Amplicon dataset**

|  | Cluster 1  (n=90) | Cluster 2 | Cluster 3 | Cluster 4 | Cluster 5 | Cluster 6 |
| --- | --- | --- | --- | --- | --- | --- |
| Cluster 2  (n=38) | 0.007 |  |  |  |  |  |
| Cluster 3  (n=67) | 0.001 | 0.50 |  |  |  |  |
| Cluster 4  (n=67) | 0.001 | 0.001 | 0.001 |  |  |  |
| Cluster 5  (n=84) | 0.001 | 0.001 | 0.001 | 0.006 |  |  |
| Cluster 6  (n=74) | 0.001 | 0.001 | 0.001 | 0.001 | 0.001 |  |
| Cluster 7  (n=66) | 0.001 | 0.001 | 0.001 | 0.001 | 0.001 | 0.37 |

**Metagenomic dataset**

|  | Cluster 1  (n=106) | Cluster 2 | Cluster 3 | Cluster 4 | Cluster 5 | Cluster 6 |
| --- | --- | --- | --- | --- | --- | --- |
| Cluster 2  (n=46) | 0.778 |  |  |  |  |  |
| Cluster 3  (n=68) | 0.186 | 0.988 |  |  |  |  |
| Cluster 4  (n=62) | 0.001 | 0.037 | 0.001 |  |  |  |
| Cluster 5  (n=76) | 0.001 | 0.001 | 0.001 | 0.001 |  |  |
| Cluster 6  (n=64) | 0.001 | 0.001 | 0.001 | 0.001 | 0.043 |  |
| Cluster 7  (n=55) | 0.001 | 0.001 | 0.001 | 0.001 | 0.001 | 0.158 |

^1^Pairwise PERMANOVA with q-values listed. q <0.25 was considered statistically significant (see Methods section). Only the earliest sample in each time cluster per infant was included in these analyses to control for multiple sampling effects.

## 2.4 Supplementary Table 4. Longitudinal comparisons^1^ of top taxa abundances and prevalences over the first 2 years of life by sequencing approach.

**Abundance**

|  | **Amplicon** (sample n=773) | **Metagenomic** (sample n=756) |
| --- | --- | --- |
| *C. albicans* | **p<0.001**, β -0.30, CI [-0.37, -0.23] | **p=0.003**, β -0.11, CI [-0.18, -0.04] |
| *Saccharomyces* | **p<0.001**, β 0.52, CI [ 0.46, 0.58] | **p=0.001**, β 0.12, CI [ 0.05, 0.19] |
| *S. cerevisiae* | **p<0.001**, β 0.14, CI [ 0.07, 0.21] | **p<0.001**, β 0.43, CI [ 0.36, 0.50] |
| *M. restricta* | **p<0.001**, β -0.29, CI [-0.36, -0.21] | **p<0.001**, β -0.16, CI [-0.24, -0.09] |
| *C. tropicalis* | p=0.69, β -0.01, CI [-0.08, 0.05] | p=0.32, β 8.63e-03, CI [-0.01, 0.03] |

**Prevalence**

|  | **Amplicon** (sample n=773) | **Metagenomic** (sample n=756) |
| --- | --- | --- |
| *C. albicans* | p=0.19, β 0.05, CI [-0.02, 0.12] | p=0.11, β -0.06, CI [-0.13, 0.01] |
| *Saccharomyces* | **p<0.001**, β 0.23, CI [ 0.16, 0.30] | **p<0.001**, β 0.23, CI [ 0.16, 0.30] |
| *S. cerevisiae* | **p<0.001**, β 0.50, CI [ 0.44, 0.56] | **p<0.001**, β 0.55, CI [ 0.49, 0.61] |
| *M. restricta* | **p=0.03**, β -0.08, CI [-0.15, -0.01] | **p<0.001**, β -0.23, CI [-0.30, -0.16] |
| *C. tropicalis* | **p<0.001**, β 0.19, CI [ 0.12, 0.26] | p=0.68, β 0.02, CI [-0.06, 0.09] |

^1^Using all samples that provided fungal sequence data; multiple samples from subjects were corrected for by including subject ID as a random effect in linear statistical models.

## 2.5 Supplementary Table 5. Comparison (*p* values) of mycobiome alpha diversity in clinical and demographic groups by time cluster and sequencing approach^1^

**Log Taxa Count (Richness)**

**Amplicon dataset**

|  | Cluster 1 | Cluster 2 | Cluster 3 | Cluster 4 | Cluster 5 | Cluster 6 | Cluster 7 |
| --- | --- | --- | --- | --- | --- | --- | --- |
| Perinatal Antibiotics | 0.71 | 0.43 | 0.52 | 0.10 | 0.88 | 0.97 | 0.44 |
| Childhood Antibiotics^2^ | N/A^3^ | 0.71 | 0.79 | 0.18 | 0.12 | 0.22 | 0.96 |
| Breastfeeding Status | 0.82 | 0.12 | 0.87 | 0.87 | 0.98 | 0.68 | 0.39 |
| Infant race^4^ | 0.20 | 0.38 | 0.92 | 0.58 | 0.48 | **0.02** | 0.29 |
| Infant sex | 0.35 | 0.59 | 0.08 | 0.40 | 0.11 | 0.61 | 0.48 |
| Birth mode | 0.27 | 0.26 | 0.12 | 0.56 | 0.76 | 0.37 | 0.28 |

**Metagenomic dataset**

|  | Cluster 1 | Cluster 2 | Cluster 3 | Cluster 4 | Cluster 5 | Cluster 6 | Cluster 7 |
| --- | --- | --- | --- | --- | --- | --- | --- |
| Perinatal Antibiotics | 0.99 | 0.85 | 0.56 | 0.26 | 0.92 | 0.81 | 0.80 |
| Childhood Antibiotics | N/A^3^ | 0.61 | **0.01** | 0.38 | **0.01** | 0.61 | 0.87 |
| Breastfeeding Status | 0.68 | 0.13 | 0.94 | 0.48 | 0.51 | 0.90 | 0.97 |
| Infant race | 0.28 | 0.71 | 0.37 | 0.75 | 0.73 | 0.11 | **0.01** |
| Infant sex | 0.66 | 0.64 | 0.56 | 0.19 | 0.20 | 0.43 | 0.83 |
| Birth mode | 0.66 | 0.45 | 0.71 | 0.45 | 0.84 | 0.42 | 0.13 |

**Shannon Diversity Index**

**Amplicon dataset**

|  | Cluster 1 | Cluster 2 | Cluster 3 | Cluster 4 | Cluster 5 | Cluster 6 | Cluster 7 |
| --- | --- | --- | --- | --- | --- | --- | --- |
| Perinatal Antibiotics | 0.78 | 0.18 | 0.54 | 0.67 | 0.27 | 0.63 | 0.70 |
| Childhood Antibiotics | N/A^3^ | 0.53 | 0.17 | 0.15 | 0.21 | 0.24 | 0.89 |
| Breastfeeding Status | 0.32 | 0.54 | 0.27 | 0.25 | 0.51 | 0.26 | 0.86 |
| Infant race | 0.57 | 0.68 | 0.42 | 0.11 | 0.95 | 0.20 | 0.22 |
| Infant sex | 0.73 | 0.91 | **0.01** | 0.74 | 0.13 | 0.65 | 0.27 |
| Birth mode | 0.32 | 0.10 | 0.66 | 0.83 | 0.36 | 0.43 | 0.51 |

**Metagenomic dataset**

|  | Cluster 1 | Cluster 2 | Cluster 3 | Cluster 4 | Cluster 5 | Cluster 6 | Cluster 7 |
| --- | --- | --- | --- | --- | --- | --- | --- |
| Perinatal Antibiotics | 0.99 | 0.39 | 0.28 | **0.04** | 0.44 | 0.87 | 0.56 |
| Childhood Antibiotics | N/A^3^ | 0.45 | 0.11 | 0.56 | 0.21 | 0.57 | 0.62 |
| Breastfeeding Status | 0.18 | 0.06 | 0.47 | 0.66 | 0.58 | 0.96 | 0.94 |
| Infant race | 0.24 | 0.66 | 0.80 | 0.26 | 0.42 | 0.08 | **0.02** |
| Infant sex | 0.61 | 0.75 | 0.97 | 0.14 | 0.19 | 0.93 | 0.46 |
| Birth mode | 0.66 | **<0.05** | 0.90 | 0.27 | 0.86 | 0.97 | **0.04** |

^1^The number of samples involved in each comparison by variable are given in Table 1.

For each comparison by primary factor, the linear statistical models were adjusted for other explanatory variables (i.e. the other factors listed in the Table). Only the earliest sample in each time cluster per infant was included in analyses to control for multiple sampling effects. *p* values < 0.05 are indicated in bold.

^2^Childhood antibiotics at the time of, or any time prior to, sample collection for each time cluster

^3^N/A, Comparison not able to be done because there were only 2 amplicon and 1 metagenomic samples exposed to antibiotics in this time cluster (out of a total of 88 and 105 samples, respectively).

^4^Comparison groups: Group 1 (non-white), Group 2 (white). See Supplemental Table 1 for breakdown of specific race groups in the study cohort

## 2.6 Supplementary Table 6. Comparison (*p*-values^1^) of abundances of predominant taxa over time in clinical and demographic groups for each sequencing approach

**Amplicon** (sample n=773)

|  | ***C. albicans*** | ***Saccharomyces*** | ***M. restricta*** | ***C. tropicalis*** |
| --- | --- | --- | --- | --- |
| Perinatal antibiotics  % exposed (55.5) | **0.01**, β 0.25, CI [0.06, 0.44]^2,3^ | **0.05**, β -0.19,  CI [-0.37, 0.00] | 0.43, β -0.07, CI [-0.23, 0.10] | 0.43, β 0.15, CI [-0.21, 0.51] |
| Childhood antibiotics  % exposed (51.1) | **0.005**, β-0.22, CI [-0.38, -0.07]^4^ | **<0.001**, β 0.47,  CI [ 0.32, 0.63] | **<0.001**, β -0.39, CI [-0.54, -0.24] | 0.75, β 0.02, CI [-0.12, 0.17] |
| Breastfeeding status  % yes (64.2) | **<0.001**, β 0.31, CI [ 0.15, 0.47] | **<0.001**, β -0.68,  CI [-0.84, -0.53] | **<0.001**, β 0.34, CI [ 0.18, 0.50] | 0.52, β 0.04, CI [-0.08, 0.15] |
| Infant race  % Group 2^5^ (74.9) | **0.006**, β -0.31, CI [-0.52, -0.09] | **0.01**, β 0.28,  CI [ 0.07, 0.49] | 0.21, β -0.12, CI [-0.32, 0.07] | 0.07, β -0.36, CI [-0.75, 0.04] |
| Infant sex  % male (56.8) | 0.34, β 0.10, CI [-0.10, 0.29] | 0.67, β 0.04,  CI [-0.15, 0.23] | 0.98, β-2.38e-3, CI [-0.17, 0.16] | 0.59, β 0.10, CI [-0.26, 0.46] |
| Birth mode  % vaginal (56.0) | **0.03**, β -0.22, CI [-0.42, -0.02]^7^ | 0.08, β 0.17,  CI [-0.02, 0.35] | 0.29, β 0.09, CI [-0.08, 0.25] | 0.42, β -0.15, CI [-0.53, 0.22] |

**Metagenomic** (sample n=756)

|  | ***C. albicans*** | ***S. cerevisiae*** | ***M. restricta*** | ***C. tropicalis*** |
| --- | --- | --- | --- | --- |
| Perinatal antibiotics  % exposed (54.6) | **0.04**, β 0.23, CI [0.02, 0.44]^8^ | 0.68, β -0.03,  CI [-0.19, 0.13] | 0.80, β 0.02,  CI [-0.15, 0.20] | 0.30, β -0.11,  CI [-0.32, 0.10] |
| Childhood antibiotics  % exposed (45.1) | 0.87, β 0.01,  CI [-0.14, 0.17] | **<0.001**, β 0.29,  CI [ 0.14, 0.44] | 0.31, β -0.08,  CI [-0.24, 0.08] | 0.96, β 9.15e-04, CI [-0.04, 0.04] |
| Breastfeeding status  % yes (71.8) | **0.01**, β 0.20,  CI [ 0.04, 0.37] | **<0.001,** β -0.55,  CI [-0.72, -0.39] | 0.11**,** β 0.14,  CI [-0.03, 0.31] | **0.02**, β -0.20,  CI [-0.37, -0.03]^6^ |
| Infant race  % Group 2^6^ (75.4) | 0.12, β -0.19,  CI [-0.43, 0.05] | 0.30, β 0.10,  CI [-0.09, 0.28] | 0.98, β 2.31e-03,  CI [-0.20, 0.20] | 0.08, β -0.22,  CI [-0.45, 0.02] |
| Infant sex  % male (55.7) | 0.11, β 0.17,  CI [-0.04, 0.39] | 0.33, β 0.08,  CI [-0.08, 0.24] | 0.19, β -0.11,  CI [-0.29, 0.06] | 0.28, β 0.12,  CI [-0.10, 0.33] |
| Birth mode  % vaginal (56.9) | **0.02**, β -0.25,  CI [-0.46, -0.03]^9^ | 0.56, β -0.05,  CI [-0.21, 0.11] | 0.78, β 0.02,  Ci [-0.15, 0.20] | 0.43, β 0.09,  CI [-0.13, 0.30] |

^1^Using all samples that provided fungal sequence data; multiple sampling of subjects was corrected for by including subject ID as a random effect in the statistical model.

^2^For each linear model comparison by primary factor, the models were adjusted for other potential explanatory variables (i.e. the other variables listed in the Table), and *p*-values remained significant except where noted.

^3^Not significant only when infant race or birth mode was added to the statistical model.

^4^Not significant only when breastfeeding status was added to the statistical model

^5^As described in Methods: Group 1, non-White; Group 2, White.

^6^Not significant only when childhood antibiotic exposure was added to the statistical model.

^7^Not significant only when breastfeeding status and perinatal antibiotic status were added to the statistical model.

^8^Not significant only when birth mode was added to the statistical model.

^9^Not significant only when perinatal antibiotic exposure was added to the statistical model.
