# Supplementary material for: Gut mycobiome maturation and its determinants during early childhood: a comparison of ITS2 amplicon and shotgun metagenomic sequencing approaches
Source: Front Microbiol. 2025 May 21;16:1539750. doi: 10.3389/fmicb.2025.1539750 (PMC12133761; doi:10.3389/fmicb.2025.1539750)
Supplement: Supplementary file 2 [file Data_Sheet_1.pdf]

Supplementary Material

1 Supplementary Figures

1.1 Supplementary Figure 1

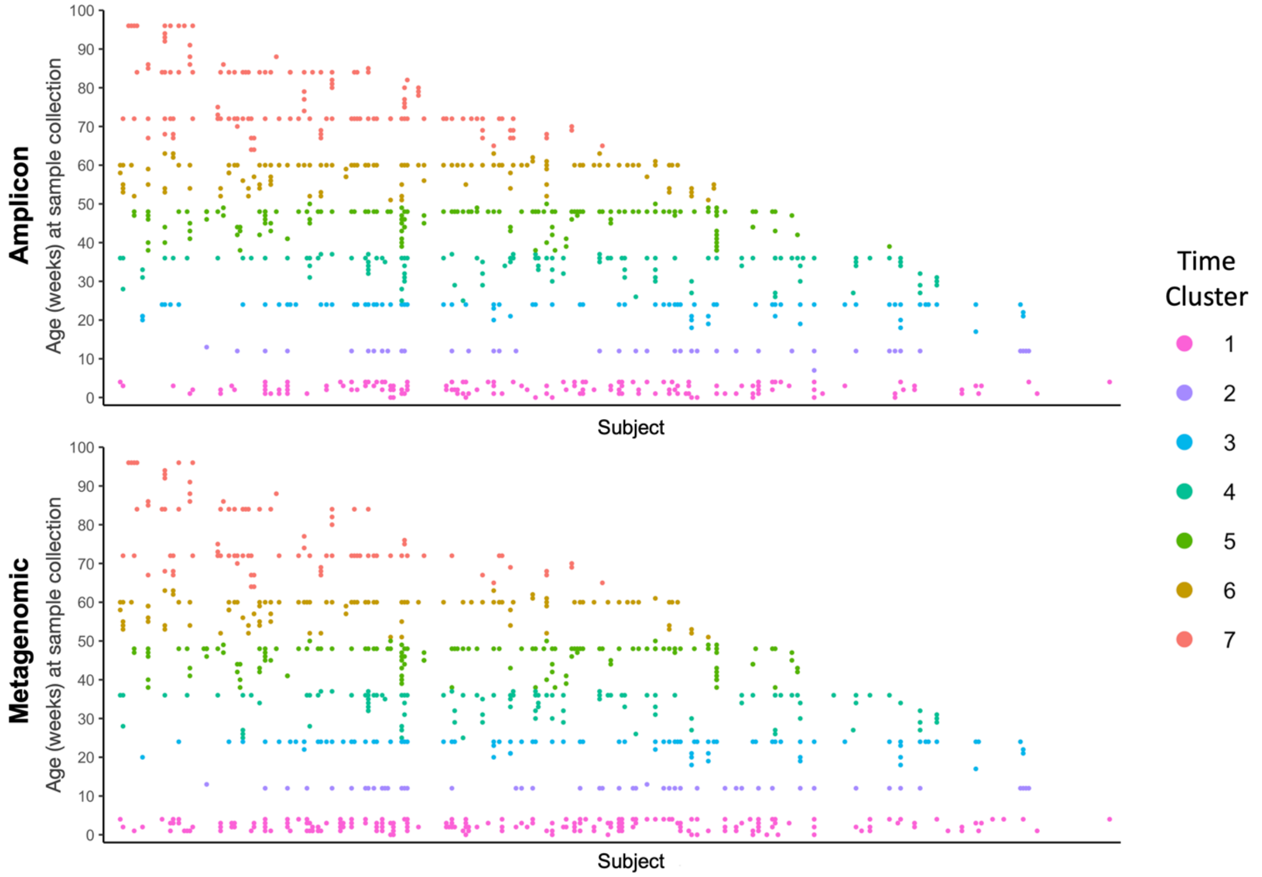

Supplementary Figure 1. Histogram of study samples by subject and age of collection. Data points are organized by subjects providing the highest number of samples on the left and those with the least number of samples on the right. Time clusters used to organize samples for longitudinal analyses are indicated by color as noted in the legend on the figure. Amplicon dataset: subject n=166 with total sample n= 773. Metagenomic dataset: subject n=173 with total sample n= 756.

1.2 Supplementary Figure 2

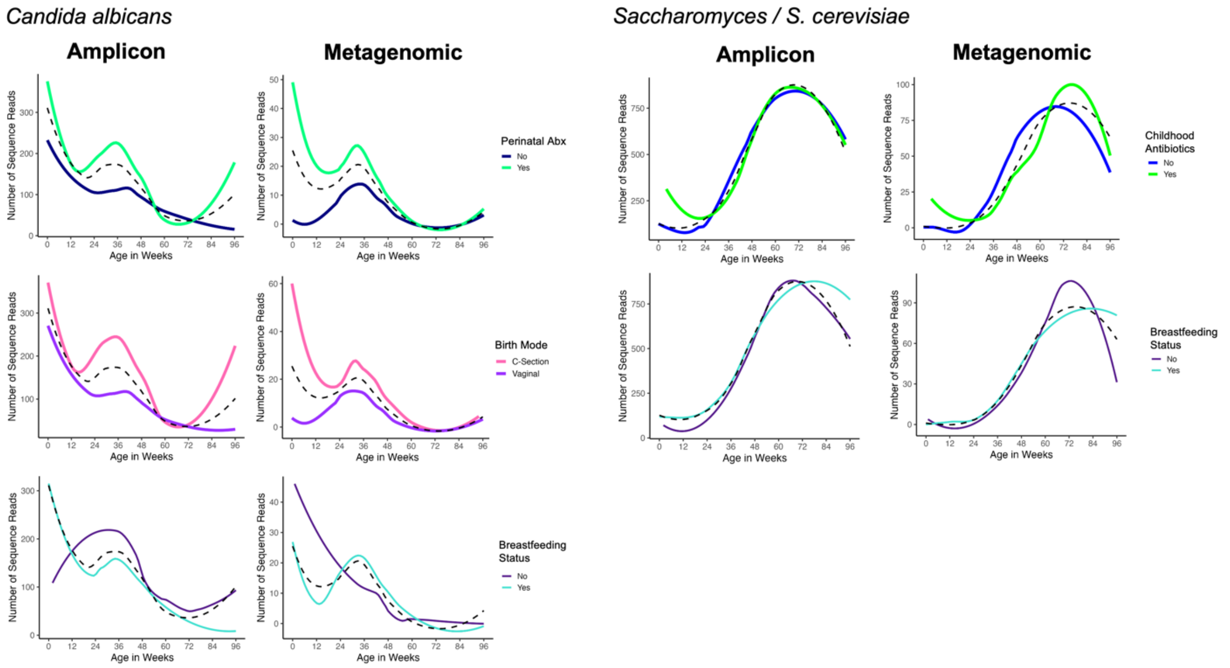

Supplementary Figure 2. LOESS regression curves of fungal taxa abundances over the first ~2 years of life in covariable subgroups for each sequencing approach. Linear statistical models were employed to compare fungal abundances over time in covariable subgroups, with *p*-values as listed in S7 table. Dotted lines represent the trends over time for the total group.
